# Vector-Borne Infections in Romania: From Surveillance to Prediction

**DOI:** 10.3390/microorganisms14010061

**Published:** 2025-12-26

**Authors:** Anca-Elena Duduveche

**Affiliations:** Department of Infectious Diseases, University of Medicine and Pharmacy of Craiova, 200349 Craiova, Romania; anca.duduveche@umfcv.ro; Tel.: +40-7-2121-9954

**Keywords:** vector-borne infections, surveillance, Romania, opportunities

## Abstract

Vector-borne infections are a growing public health concern in Romania, influenced by ecological diversity, climate change, and socioeconomic factors. West Nile virus, tick-borne encephalitis, and Lyme borreliosis represent the most significant threats, with additional risks posed by emerging pathogens, such as leishmaniasis, and the potential reintroduction of malaria. While surveillance systems exist for human cases and, to a lesser extent, vectors, these remain fragmented, underfunded, and limited in their integration across human, veterinary, and environmental health domains. By highlighting both gaps and opportunities, this review provides a forward-looking perspective on strengthening Romania’s capacity to anticipate and manage vector-borne disease threats. Transitioning from reactive surveillance to proactive, prediction-driven strategies will be critical to safeguarding public health in the context of accelerating environmental change.

## 1. Introduction

Based on recent epidemiological data and multicenter studies in Southeast Europe, the main vector-borne infections in Romania are West Nile virus (WNV) infection, tick-borne encephalitis virus (TBEV) infection, Lyme borreliosis (caused by *Borrelia burgdorferi* sensu lato), and, to a lesser extent, Crimean–Congo hemorrhagic fever virus (CCHFV). Diseases such as malaria, dengue, chikungunya, Zika, and leishmaniasis are therefore described as absent or limited to imported cases, with their inclusion justified solely by the potential risk associated with environmental change and vector expansion. The circulation of pathogens is supported by the presence of specific vectors: mosquitoes (*Culex pipiens*, *Aedes* spp.) for West Nile, and ticks (*Ixodes ricinus*) for *Borrelia* and TBE virus [1,2,3,4,5]. West Nile virus is endemic in Romania, demonstrated by its continuous presence in mosquito populations and the recurring incidence of human cases, including severe neuroinvasive forms [2,6,7]. The occurrence of severe cases is associated with both biotic and abiotic variables, with urban regions and the southeastern part of the nation facing heightened vulnerability. [6]. Lyme disease is underreported, with an under-detection rate more than 10 times that of officially reported cases, underscoring the need for increased surveillance and prevention programs [4,8].

Surveillance of vectors and vector-borne diseases is achieved through active monitoring of mosquito and tick populations, as well as the circulation of pathogens in vectors and animal hosts [3,5,9]. Recent data indicate a high diversity of vector species and a significant prevalence of co-infections in urban environments [3]. Prediction of the risk of outbreaks is based on epidemiological models that integrate climatic, ecological, and vector mobility data [6].

Climate change, globalization, and urbanization are increasing the risk of vector-borne diseases in Europe by expanding the range and season of activity of vectors (mosquitoes, ticks, sandflies), and favoring the autochthonous transmission of pathogens such as West Nile virus, Dengue, Chikungunya, Zika, Lyme disease, and tick-borne encephalitis. Rising temperatures, changing rainfall patterns, and milder winters are enabling vectors to survive and multiply in previously unaffected areas, including at higher altitudes and latitudes. Meanwhile, land-use changes and habitat fragmentation are exacerbating human exposure [10,11,12,13,14].

Globalization, by increasing international transport of people and goods, facilitates the introduction and dispersal of invasive vectors (for example, *Aedes albopictus* and *Aedes aegypti*) and pathogens, reducing geographic barriers and accelerating the emergence of new outbreaks [14,15,16]. Urbanization creates microhabitats conducive to vectors (water accumulations, increased human density), favoring transmission and making control difficult [16].

Romania presents remarkable ecological diversity, with ecosystems such as the Danube Delta, the Carpathian Mountains, and agricultural plains, which generate a mosaic of habitats for vectors. The Danube Delta offers optimal conditions for the development and diversification of mosquito populations (*Coquillettidia richiardii*, *Anopheles hyrcanus*, *Culex pipiens*), and is recognized as a major hotspot for West Nile virus and other arboviruses. The Carpathian Mountains and adjacent forest areas support populations of ticks (*Ixodes ricinus*), vectors for *Borrelia*, TBEV, and other zoonoses, with a high diversity of wild hosts. Agricultural plains and peri-urban areas facilitate the coexistence of vectors and hosts, amplifying the risk of transmission to humans, especially in the context of climate change and the intensification of agricultural activities [2,5,17,18].

Surveillance has historically been reactive, based on human case notification and ad hoc entomological surveys, with incomplete geographical coverage and a lack of integration of data from multiple sources [5,17,19]. There is a clear need for predictive approaches and the modernization of surveillance infrastructure.

The objective of this review is to provide a comprehensive overview of the current epidemiology of vector-borne infections in Romania with emphasis on West Nile virus, tick-borne encephalitis, Lyme disease, and other emerging threats. This review also evaluates the performance of existing surveillance approaches, including case reporting, entomological monitoring, and laboratory diagnostics. Moving beyond surveillance, this review explores predictive tools that could transform early warning and response capacity: climate and environmental modeling to anticipate vector expansion, genomic epidemiology to trace pathogen diversity and transmission, and digital platforms that enable real-time data collection and citizen engagement. It also exposes a One Health approach that bridges medical, veterinary, and ecological data, particularly in cross-border collaborations within Eastern Europe. By integrating One Health and interdisciplinary approaches, this highlights the opportunities to strengthen preparedness at the human–animal–environment interface and also to outline a roadmap for transitioning from reactive surveillance to proactive, prediction-driven strategies in Romania.

## 2. The Main Vector-Borne Infections in Romania

### 2.1. West Nile Virus (WNV)

West Nile virus is a positive-sense, single-stranded RNA virus belonging to the *Flavivirus* genus and the Flaviviridae family [10]. Approximately 80% of infections are asymptomatic. Symptomatic cases typically present as West Nile fever, with symptoms such as fatigue, fever, headache, muscle weakness, and occasionally rash or gastrointestinal symptoms [10].

The epidemiology of West Nile virus (WNV) infection in Romania is characterized by endemic circulation and recurrent outbreaks, with a predominant geographical distribution in the southeast of the country and urban areas, including Bucharest [7,19]. The first major outbreak was reported in 1996, with 393 confirmed cases and 17 deaths [20]. Significant epidemics followed in 2010, 2016, and 2018, these being associated with the transmission of lineage 2 of the virus, which gradually replaced lineage 1 and shows a dynamic genetic evolution with multiple sub-lineages and clusters [21,22]. The primary animal reservoirs are wild and domestic birds, with particular emphasis on resident Passeriformes and domestic fowl. The main vectors are *Culex pipiens* (dominant urban and periurban vector) and the emerging *Aedes albopictus*, recently detected in urban areas and implicated in WNV transmission [23]. The virus circulates in an enzootic cycle between birds and mosquitoes, with humans being accidental terminal hosts; other less frequent transmission methods include blood transfusion, organ transplantation, and maternal-to-child transmission [24].

Bird migration plays a critical role in the dissemination of WNV in Romania. Phylogenetic analyses of WNV strains isolated in Romania reveal close genetic relationships with strains from Africa and other parts of Europe, supporting the hypothesis that migratory birds introduce new viral lineages into the region [10]. The Danube Delta, a major stopover for migratory birds, is a key ecological hotspot for WNV introduction and maintenance. The spatial structure of outbreaks in Romania and across Europe is strongly associated with migratory bird flyways and wetland habitats, which are repeatedly identified as drivers of WNV spread and genetic diversity.

The surveillance approach includes seasonal monitoring of mosquito populations (captures, molecular testing for WNV) and reporting of human cases, with a focus on neuroinvasive forms [6,25]. Entomological surveillance is focused on high-risk areas, and clinical data come from notification of hospitalized cases. The main challenges are the underreporting of asymptomatic infections (most WNV infections are subclinical or with mild symptoms, so they do not reach the reporting system) and the late detection of cases, which limits the capacity to respond rapidly to outbreaks [6,26]. The integration of entomological, clinical, and laboratory data is insufficient, and reactive surveillance does not allow for the anticipation of epidemics. Thus, WNV remains the main vector-borne infection in Romania, with dynamic circulation, multiple vectors, seasonal surveillance, and major challenges related to underreporting and late detection.

### 2.2. Tick-Borne Encephalitis (TBE)

Tick-borne encephalitis (TBE) virus is classified as a member of the genus *Flavivirus*, family Flaviviridae. The epidemiology of TBE in the endemic regions of northwestern and central Romania is characterized by focal circulation of the TBE virus, with confirmed presence in both sentinel animals (e.g., sheep) and humans. Recent serological studies have identified a seroprevalence of anti-TBEV antibodies of 0.08% in healthy blood donors in northwestern Romania, indicating a low, but existing, human exposure in the general population [27]. In contrast, the prevalence of antibodies in sheep from the same areas is much higher (15.02%), suggesting active circulation of the virus in rural and mountain ecosystems, with persistent natural foci [27,28].

The main vector is *Ixodes ricinus*, which predominates the forest and hilly regions of the northwest and center, with peak seasonal activity from April to November [29,30]. Transmission to humans occurs predominantly by tick bite, but isolated cases associated with consumption of unpasteurized milk from infected animals have been reported [31,32]. The incidence of human cases is underestimated due to the lack of active surveillance and diagnostic difficulties, with most cases being sporadic and associated with recreational or occupational activities in endemic areas [33,34]. The principal clinical features include a biphasic illness in most symptomatic cases. The first phase presents as a nonspecific febrile illness with symptoms such as fever, malaise, headache, myalgia, and sometimes gastrointestinal symptoms. After a brief asymptomatic interval, the second phase involves the central nervous system and typically presents as aseptic meningitis, encephalitis, or meningoencephalomyelitis. Neurological findings may include altered mental status, ataxia, cranial nerve palsies, limb paresis, seizures, and tremors. Severity increases with age, and long-term sequelae or post-encephalitic syndrome occur in a substantial proportion of cases. Most infections are asymptomatic, but severe neuroinvasive disease can result in significant morbidity and, depending on viral subtype [32].

Low clinical suspicion stems from the low reported incidence and lack of awareness of TBE among physicians, leading to underdiagnosis, especially in cases with nonspecific presentation or subclinical forms [27,33]. Initial symptoms may be similar to other viral infections, and the neuroinvasive phase occurs in 20–30% of patients, requiring a detailed history of tick exposure [35].

Limited serological testing is a major barrier: access to ELISA and neutralization tests for anti-TBEV antibodies is restricted, and serum collection is not standardized according to the time of symptom onset, which reduces diagnostic sensitivity [36]. Also, co-circulation of other flaviviruses (WNV, USUV) complicates serological interpretation due to cross-reactions [37].

Vaccination is available but not routinely practiced, unlike in Austria, where vaccination coverage > 80% has dramatically reduced the incidence of TBE. In Romania, the lack of strong recommendations and information campaigns leads to insufficient protection of at-risk groups [32,33]

Increased exposure to vectors is driven by the increase in rural tourism and forestry activities, with seasonal risk peaking in spring and summer [32]. This phenomenon amplifies the number of exposed people, including tourists and forestry workers, who are unaware of the TBE risk and do not seek testing or prophylaxis [38].

### 2.3. Lyme Disease

Lyme borreliosis is caused by spirochetes in the *Borrelia burgdorferi* sensu lato complex, which are bacteria in the genus *Borrelia* [39,40,41]. The primary transmission route is the bite of infected *Ixodes* ticks. It is essential to note that Lyme borreliosis is caused by several genospecies within the *Borrelia burgdorferi* sensu lato complex, most notably *Borrelia burgdorferi* sensu stricto, *Borrelia afzelii*, and *Borrelia garinii*. These are spirochetal bacteria in the genus *Borrelia*, and the specific species involved can influence the clinical spectrum, with *B. burgdorferi* sensu stricto more commonly associated with arthritis (especially in North America), *B. garinii* with neurologic involvement, and *B. afzelii* with cutaneous manifestations such as acrodermatitis chronica atrophicans, particularly in Europe [39,41].

The epidemiology of Lyme disease (Lyme borreliosis) in the Carpathian region, considered a hotspot for the *Ixodes ricinus* tick, is marked by an increased incidence and fragmented collection of epidemiological data. Lyme borreliosis is the most common vector-borne disease in Europe, and in the Carpathians, the high density of forests, the biodiversity of hosts (rodents, cervids, birds), and the temperate climate favor the maintenance of abundant populations of *Ixodes ricinus*, with maximum activity in spring and autumn [39,40].

The prevalence of infection with *Borrelia burgdorferi* sensu lato in *Ixodes ricinus* in Romania is high, with infection rates of approximately 18% at the national level. In the Carpathian areas, the prevalence may be even higher, similar to data from Slovakia and Poland, where values of 20–30% in nymphs and adults are reported [41,42]. The main genospecies identified are *B. afzelii* (predominant), *B. garinii,* and *B. valaisiana*, with local variations determined by the host spectrum and habitat type [40,42,43]. Coinfections with other pathogens (e.g., *Babesia* spp.) are reported in 3–10% of ticks, which can complicate the clinical picture [44,45].

The clinical incidence of Lyme borreliosis is significantly underestimated: for every officially reported case, there are over 10 unreported symptomatic cases, according to seroprevalence studies in Romania [8,46]. Fragmentation of the surveillance system, lack of standardization of case definitions and diagnostic procedures, and regional variability in reporting contribute to underdiagnosis and difficulties in estimating the true risk [47].

The symptomatic overlap between Lyme borreliosis and autoimmune or rheumatological diseases leads to a high rate of misdiagnosis in the Carpathian region, as clinical manifestations such as arthralgia, myalgia, fatigue, and skin phenomena can mimic lupus, scleroderma, dermatomyositis, or rheumatoid arthritis. The mechanisms of molecular mimicry and persistent immune response induced by *Borrelia burgdorferi* complicate clinical differentiation, and the lack of strict diagnostic criteria favors confusion between infection and autoimmune pathology [48,49,50]. This phenomenon determines the initiation of inadequate treatments, delay in antimicrobial therapy, and increased risk of chronic complications.

The involvement of private laboratories with unregulated and inconsistent reporting affects the accuracy and consistency of epidemiological data collection, as the use of unvalidated serological methods, inconsistent test interpretation (e.g., false positive IgM, lack of standardized two-stage testing algorithm), and lack of centralized reporting leads to overdiagnosis or underdiagnosis, fragmenting the true picture of incidence [8,51,52]. Discrepancies between laboratories regarding the sensitivity and specificity of tests, as well as the lack of mandatory reporting of results to public health authorities, generate underestimation or overestimation of the true incidence and prevent effective monitoring of epidemiological trends [53,54].

### 2.4. Other Vector-Borne Infections of Concern

#### 2.4.1. Leishmania

Protozoan parasites of the genus *Leishmania* cause leishmaniasis. The transmission route is via the bite of infected female sandflies. Principal clinical features depend on the infecting species and host factors, and include three primary syndromes: cutaneous, visceral, and mucosal leishmaniasis. Historically, Romania has been considered non-endemic for human leishmaniasis, with only sporadic cases reported over several decades. The risk of imported leishmaniasis in the Carpathian region is increased due to increased travel to endemic areas in southern Europe and the Mediterranean, with recent documented cases in people returning from Spain and Italy, including children and adults, according to current medical literature [55]. Diagnosis is often delayed, and the clinical presentation varies, requiring increased suspicion in patients with chronic skin lesions and travel history.

According to climatic and ecological models, there is a real potential for local establishment of leishmaniasis in the Carpathians. Climate change favors the northward expansion of competent vectors (*Phlebotomus* spp.), with increasing temperatures and changing precipitation regimes, making areas in central and northwestern Romania increasingly favorable for vector survival and reproduction [56,57,58]. Ecological modeling studies show that by 2050, and even more markedly by 2070–2080, the Carpathians may develop climatic conditions similar to Mediterranean areas, creating an environment suitable for Leishmania vector establishment. This highlights the realistic possibility of future autochthonous outbreaks if parasites are introduced [56,57].

The risk of local establishment depends on the presence of vectors, the introduction of the parasite, and the existence of reservoir hosts (dogs, rodents). Once vectors are established and the parasite is introduced, local transmission becomes possible, with the potential for the appearance of visceral and cutaneous forms, especially in vulnerable populations [59,60]. A 1 °C increase in temperature is associated with a higher incidence of leishmaniasis in areas of southeastern Europe [61].

#### 2.4.2. Crimean–Congo Hemorrhagic Fever (CCHF)

Crimean–Congo hemorrhagic fever virus (CCHFV) is classified as a negative-sense, single-stranded RNA virus in the genus *Orthonairovirus*, family Nairoviridae, order Bunyavirales [62]. The CCHF virus is mainly transmitted by ticks of the genus *Hyalomma*, and these species are already established in southern Romania [63]. Serological surveys of small ruminants in the area have shown a high seroprevalence of over 35%, indicating active viral circulation in animals and a consequent potential exposure risk for humans [62]. The principal clinical features include abrupt onset of high fever, severe headache, myalgia, dizziness, and gastrointestinal symptoms (nausea, vomiting, diarrhea). The disease may progress to hemorrhagic manifestations such as petechiae, ecchymoses, epistaxis, hematuria, and gastrointestinal bleeding [62].

The risk of Crimean–Congo hemorrhagic fever (CCHF) spread in Romania is considered significant, given the regional epidemiological context and current ecological factors.

Neighboring Balkan countries (Bulgaria, Serbia, Albania, Greece, Turkey) have reported confirmed human cases and recurrent outbreaks, with the expansion of the *Hyalomma* vector range favored by climate change, animal migration, and intensification of agricultural activities [63,64,65]. Phylogeny and seroprevalence studies show that the virus circulates in Southeastern Europe, and Romania is in the epidemiological risk zone, with the potential for the emergence of autochthonous human cases, especially in the south and southeast [66,67]. Importation of animals, bird migration, and increasing temperatures favor the expansion of vectors and the virus to new areas, including Romania [68,69]. There is no approved vaccine or specific antiviral treatment, and underdiagnosis is common due to subclinical forms in humans [70].

#### 2.4.3. Malaria

Malaria is caused by protozoan parasites of the genus *Plasmodium*. The principal transmission route is the bite of an infected female Anopheles mosquito. The principal clinical features are fever, chills, headache, myalgias, and malaise, often accompanied by gastrointestinal symptoms such as vomiting and diarrhea.

The risk of malaria reintroduction in Romania is considered low, but not negligible, given the European epidemiological context and local factors. Although malaria was officially eradicated in Romania in 1963, current cases are exclusively imported, occurring in travelers or migrants from endemic areas, especially Africa and Asia, with over 20–30 imported cases annually [71,72,73]. Neighboring countries in Central and Eastern Europe similarly report only sporadic imported cases, with rare instances of locally acquired malaria, typically classified as Odyssean or induced malaria, rather than sustained transmission cycles. Surveillance efforts in these regions focus on rapid case identification, vector monitoring, and public health infrastructure to prevent re-establishment of endemic malaria. High-resolution spatial modeling and recent global surveillance data confirm that the region remains non-endemic, but highlight the importance of ongoing vigilance due to environmental and demographic changes [74].

Anopheles vectors (*An. maculipennis s.l*., *An. hyrcanus*, *An. atroparvus*, *An. messeae/daciae*) are still widely distributed in Romania, including in the Danube Delta and agricultural areas, and favorable climatic conditions (high temperatures, humidity) may support the transmission cycle of *Plasmodium*, especially in the context of climate change [5,74,75,76]. However, autochthonous transmission has not been documented in recent decades, due to the efficiency of the public health system, early diagnosis, and rapid treatment of imported cases.

The risk of reintroduction is exacerbated by increased international mobility, migration from endemic areas, lack of prophylaxis in travelers, and the presence of competent vectors. Predictive models indicate that, in the absence of active surveillance and vector control, reintroduction of malaria is possible, especially in areas with high *Anopheles* densities and exposure to imported cases [77,78].

## 3. Current Surveillance Systems in Romania

### 3.1. Reporting Human Cases

The characteristics of the current epidemiological surveillance systems in Romania include a centralized structure, with reporting of human cases of notifiable diseases through the National Institute of Public Health (INSP). This system is based on mandatory notification of cases by medical units, with data collection at the county level and their transmission to the INSP, where they are aggregated, analyzed, and reported at the national and international levels [8,79]. The efficiency of surveillance is affected by underreporting, data fragmentation, and variability in the quality of reporting between counties. For Lyme borreliosis, for example, it has been shown that the true incidence is more than ten times higher than that officially reported, due to underdiagnosis and the lack of integration of data from private laboratories. In the case of measles, surveillance has allowed the rapid identification of outbreaks and viral variants, but the performance of the system is limited by insufficient vaccine coverage and the variable quality of epidemiological data [80].

The current system allows monitoring of epidemiological trends, identifying outbreaks and implementing control measures, but there are needs for modernization, integration of alternative data sources (electronic records, genomic surveillance, wastewater) and increased interoperability with European systems, according to the strategic directions discussed at the EU/EEA level [81].

### 3.2. Entomological Surveillance

Current entomological surveillance methods in Romania include seasonal capture of mosquitoes with specialized traps (e.g., carbon dioxide traps, ovitraps), morphological and genetic identification, and molecular testing for pathogens. In Bucharest, continuous monitoring of *Culex pipiens* and *Aedes albopictus* populations is performed annually, with frequent detection of West Nile virus in *Culex pipiens* and identification of circulating viral sub-lineages, reflecting a dynamic and complex circulation of WNV [2,82]. Efficiency is high for the detection of the main vectors and the virus, but geographical coverage is limited to urban and peri-urban areas at high risk.

In the Danube Delta, longitudinal surveillance with carbon dioxide traps allowed the collection of a very large number of mosquitoes (over 240,000 females in one season), with the dominant identification of *Coquillettidia richiardii* and *Anopheles hyrcanus*, important vectors for WNV. The studies highlighted a high diversity of species, including two new to Romania, and integrated genetic analysis (DNA barcoding) for species confirmation [17,83,84]. The efficiency is very good for characterizing local fauna and assessing the risk of transmission, but surveillance is not permanent; it is carried out in specific projects.

Tick mapping is done sporadically, through academic projects and initiatives of local public health authorities, with manual collection from animals and the natural environment, followed by morphological identification and molecular testing for pathogens (*Borrelia, Rickettsia, Anaplasma*). Data are fragmented, geographical coverage is incomplete, and mapping at the national level is carried out mainly through international collaborations (e.g., VectorNet), which integrate data from the scientific literature and from the field [18,29,85].

### 3.3. Laboratory Capacity

Current laboratory capacity for vector-borne disease surveillance in Romania includes the routine availability of PCR (real-time and conventional) and ELISA for WNV and Lyme borreliosis in university centers, public health institutes, and some private laboratories. PCR is used for the direct detection of WNV viral RNA in blood, cerebrospinal fluid, or mosquito pools, with validation by viral isolation on cell lines, being the standard for confirmation of acute infection and for vector surveillance [1,2,83]. For *Borrelia burgdorferi* sensu lato, PCR is used for the detection of DNA from tissue samples, synovial fluid, or ticks, with increased sensitivity in early stages [40].

ELISA is the primary method for detecting IgM and IgG antibodies to WNV and *Borrelia*, both in human diagnosis and in seroprevalence studies in blood donors and sentinel animals [27,86]. The serological algorithm for Lyme includes ELISA testing followed by Western blot confirmation, according to European practice [4]. Major limitations are related to unequal access to advanced serological testing (e.g., viral neutralization, multi-antigen microarray), lack of standardization of collection timing and interpretation of results, and cross-reactions between flaviviruses [4,87]. Genetic sequencing for tracking WNV and Borrelia outbreaks is available in reference laboratories, but capacity is limited by cost, infrastructure, and access to representative samples. Recent studies have highlighted difficulties in obtaining complete sequences from clinical samples with low viral load, data fragmentation between laboratories, and the lack of an integrated national database for phylogeny and molecular surveillance [2,20]. This restricts the ability to rapidly track viral evolution and identify the source of outbreaks.

### 3.4. Gaps

The main gaps identified in the epidemiological and entomological surveillance of vector-borne diseases in Romania are: underreporting of cases in rural areas, the lack of integrated databases that include information on humans, vectors, and animals, and the existence of a small number of sentinel animal surveillance programs.

Underreporting of cases in rural areas is documented for both WNV infections and tick-borne encephalitis and Lyme borreliosis, due to limited access to diagnosis, low clinical suspicion, and fragmentation of the reporting system, which leads to an underestimation of the true incidence [6,27,88]. Most reported cases come from urban areas or university centers, and asymptomatic or mild forms are not captured by the current system.

The lack of integrated databases linking human, vector, and animal information hinders comprehensive epidemiological analysis and outbreak prediction. Entomological, clinical, and veterinary data are collected separately, without interoperability, which limits the ability to rapidly identify high-risk areas and implement effective control measures [17,83]. There is no national platform integrating laboratory data, vector surveillance, and results from sentinel animals.

Sentinel animal surveillance programs (horses, birds) for WNV monitoring are sporadic and limited to ad hoc projects, without national coverage or continuity. Studies in the Danube Delta and the southeast of the country have shown high seroprevalence in birds and horses, but these data are not systematically collected and are not integrated into routine epidemiological surveillance [7]. The absence of continuous monitoring of sentinel animals reduces the capacity for early warning and anticipation of outbreaks.

These gaps affect Romania’s ability to detect vector-borne diseases early and effectively control them, requiring investments in surveillance infrastructure, data integration, and expansion of sentinel animal monitoring programs.

## 4. From Surveillance to Prediction

### 4.1. Climatic and Environmental Data

Increasing average temperatures and longer summers extend the transmission season of vector-borne diseases by extending the period of activity of mosquitoes (*Culex pipiens*, *Aedes albopictus*, *Anopheles* spp.) and ticks (*Ixodes ricinus*), accelerating the development cycle of vectors and increasing the rate of reproduction, leading to higher density and an extended epidemiological window for the transmission of pathogens such as West Nile virus, *Borrelia*, and TBEV [89,90]. High temperatures favor viral replication in vectors and shorten the extrinsic incubation period, increasing the risk of outbreaks, as observed in Bucharest and the Danube Delta [2].

Heavy rains and floods, especially in the Danube Delta area, favor the proliferation of mosquitoes and increase the risk of vector-borne disease transmission by creating new temporary and permanent aquatic habitats, which serve as oviposition and larval development sites for dominant vector species (e.g., *Coquillettidia richiardii*, *Anopheles hyrcanus*, *Culex pipiens*) [91]. Floods cause a rapid increase in the density of mosquito populations, especially floodwater species (*Aedes vexans*, *Aedes caspius*), which can generate peaks of activity 1–4 weeks after the pluvial event [92]. The accumulation of stagnant water after floods extends the period of vector activity and increases the risk of transmission of West Nile virus, heartworm disease, avian malaria, and other vector-borne diseases, both to humans and animals [93]. Studies in the Danube Delta have shown that these conditions favor the maintenance and diversification of vector populations, with the potential for zoonotic and human outbreaks [93]. The epidemiological risk is amplified by interaction with reservoir hosts (birds, horses, dogs) and by the disruption of local ecosystems.

Land-use changes, such as deforestation and irrigation, significantly alter tick habitat and influence the transmission of vector-borne diseases. Deforestation initially reduces forest habitat, decreasing tick density, but subsequent forest fragmentation favors recolonization by key hosts (e.g., deer, rodents), leading to increased populations of *Ixodes ricinus* and the spread of diseases such as Lyme disease and tick-borne encephalitis [94,95]. Habitat fragmentation and changes in predator communities increase the abundance of small mammals, which are effective hosts for pathogens, increasing the risk of transmission to humans [96]. Irrigation and agricultural expansion alter the local microclimate, increasing soil moisture and vegetation, favoring tick survival and development [97]. Agricultural and irrigated areas can support high densities of ticks, especially at the edge of forests or near habitats with wild and domestic hosts. The diversity of the agricultural landscape influences the distribution of tick species and associated pathogens, with variable risks to public health [98].

### 4.2. Predictive Models

The predictive models used to model the ecological niche of *Culex* mosquitoes and *Ixodes* ticks in Europe are based on ecological niche modeling (ENM) and species distribution modeling (SDM), with algorithms such as MaxEnt (maximum entropy), random forest, generalized additive models (GAM) and machine learning methods (e.g., gradient boosting, neural networks) [99,100,101]. These models integrate presence/absence data from validated databases (VectorNet, ECDC), bioclimatic variables (temperature, precipitation, humidity), land cover, habitat fragmentation, vegetation, host presence, and anthropogenic factors [9,102].

The maps produced by the European Centre for Disease Prevention and Control (ECDC) and the VectorNet project are considered the current standard for vector mapping at the European level, using aggregated data from scientific literature, field collections, and expert validation [102]. For *Culex pipiens*, SARIMA models and empirical regressions on time series of catches correlated with meteorological variables (temperature, humidity) are used to predict seasonal abundance [103]. For *Ixodes ricinus*, MaxEnt and random forest models integrate climate and land cover data, but performance is increased when local variables (vegetation type, host density, habitat fragmentation) are added [101].

The most recent projects in Romania that applied predictive models to model the ecological niche of *Culex* mosquitoes and *Ixodes* ticks have integrated local climate and land cover data, but remain limited in resolution and national coverage. For mosquitoes, a synthesis published in 2020 generated a georeferenced database with the distribution of *Culex* species, using data from local literature and field collections, with GIS mapping, but without advanced integration of climate or land cover variables at the level of predictive modeling. In the Danube Delta, longitudinal studies used seasonal captures and genetic analysis for species identification, highlighting correlations between vector abundance and climate parameters, but without the development of complex predictive models [5,17].

For *Ixodes ricinus*, Romanian projects have contributed presence and abundance data to European collaborations such as VectorNet, which uses MaxEnt, random forest, and generalized additive models, integrating climate and land cover variables at the continental level [9]. European models, including the ECDC maps and VectorNet, offer sub-national resolution, expert validation, and integration of data from multiple sources, and are considered the current standard for predicting vector distribution in Europe [9]. These models include data from Romania, but are not optimized for local climate and land cover, which may limit the accuracy of regional predictions [99].

Compared to European collaborations, Romanian models are more fragmented, with incomplete geographical coverage and limited integration of local variables, while VectorNet and ECDC offer validated maps, an extensive data archive, and standardized methodology, being superior for large-scale risk assessment [9,99]. There is a clear need to develop predictive models specific to Romania, integrating microclimate, land cover and local abundance data to optimize vector surveillance and control.

### 4.3. Genomic and Molecular Approaches

For the complete sequencing of the WNV genome in Romania, current approaches include multiplex PCR followed by NGS (Next Generation Sequencing) sequencing, with platforms such as Illumina or Oxford Nanopore, directly from biological samples (blood, tissues, mosquito pools), without the need for viral isolation on cell cultures [104]. The multiplex PCR protocol allows coverage of the entire viral genome even from samples with medium viral load (Ct ≈ 30), facilitating phylogenetic analysis and identification of transmission lineages, including sub-lineages 2a and 2b, as well as local clusters and multiple introductions from neighboring countries. In laboratories with limited infrastructure, partial sequencing of structural genes (NS5, E, NS3) by conventional PCR and Sanger, followed by phylogenetic analysis for rapid strain classification, is frequently used [1,22,105].

For genotyping *Borrelia burgdorferi* sensu lato, multiplex PCR (qPCR) on the ospA and flaB genes is applied, followed by conventional sequencing and identification of genospecies (*B. afzelii, B. garinii, B. valaisiana, B. miyamotoi*) [40,106]. For the assessment of strain diversity, MLST (multilocus sequence typing) on 8 house genes is recommended, but this method is rarely implemented due to limited infrastructure and costs. In practice, most public laboratories use PCR and Sanger sequencing for genospecies identification, with limited integration of data into national databases [107].

Major gaps include: low capacity for whole genome sequencing (WGS) due to cost, insufficient IT infrastructure and bioinformatics expertise, data fragmentation across laboratories, and the lack of an integrated platform for national genomic surveillance [108,109,110]. This limits monitoring of viral and bacterial evolution, as well as the ability to rapidly track transmission and strain diversity in regional outbreaks.

### 4.4. Digital Instruments

The current status of digital tools for vector surveillance in Romania is emerging, with significant potential for expansion. Syndromic surveillance applications piloted during the COVID-19 pandemic have demonstrated that mobile reporting can accelerate epidemiological data collection and increase the accuracy and speed of outbreak response, including for vector-borne diseases, by integrating symptom notifications with laboratory data and public health systems [111,112].

Citizen science initiatives, such as the global platforms iNaturalist or Mosquito Alert, allow the reporting of mosquito and tick observations by the population, with expert validation of images and location, generating georeferenced databases useful for mapping vector distribution and identifying areas of expansion or risk [113,114,115]. These data can complement traditional entomological surveillance, rapidly covering unexplored areas and facilitating early detection of invasive species or seasonal changes [115].

Integrating mobile reporting with laboratory confirmation allows for real-time mapping of vector distribution, correlating field observations with molecular data (PCR, ELISA) and validated maps from European networks such as VectorNet [9]. This model reduces data fragmentation, optimizes resource allocation, and allows for rapid interventions in areas of high epidemiological risk.

## 5. One Health Perspective

The critical zoonotic interface represents the ecological and epidemiological contact point between animal hosts, vectors, and humans, and is essential in the One Health perspective for the control and surveillance of vector-borne diseases such as West Nile virus (WNV) and Lyme disease.

For West Nile virus, birds are the main reservoirs and amplifiers of the virus, supporting viral circulation in the ecosystem via mosquitoes (*Culex* spp.). Horses and humans are dead-end hosts, accidentally infected, with no role in maintaining the viral cycle, but at risk of severe neuroinvasive disease. Integrated surveillance of birds (including serological and molecular monitoring), vectors, and horses allows for early detection of WNV circulation and anticipation of the risk to humans, being a pillar of the One Health approach [116,117,118].

For Lyme disease, rodents (e.g., mice, squirrels) are effective reservoirs for *Borrelia burgdorferi* sensu lato, providing infection to *Ixodes ricinus* ticks. Deer and moose are not reservoirs for *Borrelia*, but they support adult tick populations, amplifying the risk of human exposure and vector dispersal [119,120]. The zoonotic interface is determined by the density and movement of these hosts, habitat fragmentation, and local biodiversity, which influence the prevalence of infection in vectors and the risk to humans.

The One Health approach requires integrated surveillance of reservoir animals, vectors, and humans, correlation of ecological and epidemiological data, and coordinated interventions to reduce zoonotic risk [121]. Protecting biodiversity and managing animal habitats are key strategies to limit pathogen amplification and the risk of transmission to humans. To provide a Romania-specific, integrative overview, Table 1 synthesizes key epidemiological signals, vectors and reservoirs, geographic risk areas, surveillance gaps, and priority predictive solutions for the major vector-borne diseases addressed in this review.

### 5.1. Concrete Integrated Surveillance Strategies

Strategies that have been shown to be effective in reducing the risk of WNV and Lyme disease transmission within the One Health approach include:

- Coordinated surveillance of vectors, reservoir animals, and human cases: Simultaneous surveillance of mosquitoes (seasonal captures, molecular testing for WNV), birds, and horses (serology, PCR), as well as human cases, allows for early detection of viral circulation and rapid activation of control measures. Integrating data from these sources reduces response time and optimizes interventions [122].
- Use of sentinel animals: Serological surveillance of birds and horses for WNV, and rodents for *Borrelia*, respectively, provides early indicators of pathogen circulation and allows for prediction of risk to humans [123].
- Establishing formal interagency groups: Collaboration between public health authorities, veterinarians, and entomologists, with data exchange and common protocols, has proven essential for the efficiency of integrated surveillance and for saving resources [124].
- Implementing early warning systems: Integrating entomological, veterinary, and human data into digital platforms, with prediction algorithms and risk maps, allows for the rapid activation of vector control measures and information of the population [90].
- Active surveillance of vectors and hosts: Periodic capture and testing of mosquitoes and ticks, correlated with monitoring of reservoir animals, provides a complete picture of the epidemiological risk and allows for targeted interventions [116].
- Intersectoral communication and education: Information campaigns for professionals and the public, based on integrated data, increase acceptance of prevention measures and reduce exposure to vectors [125].

These approaches have proven effective in Europe and the US, reducing costs and improving outbreak control, according to One Health studies and analyses.

### 5.2. Regional Collaboration

Bulgaria, Hungary, and Serbia have fostered regional collaboration to facilitate cross-border data exchange for integrated surveillance of vector-borne zoonotic diseases, such as West Nile virus and Lyme disease. These strategies have demonstrated effectiveness in increasing the capacity for early detection, rapid response, and control of vector-borne diseases at the regional level, according to recent European literature, and include [36]:

- Establishing formal intersectoral working groups (One Health), involving public health authorities, veterinarians, entomologists, and animal health specialists, to coordinate surveillance and data exchange at the regional level [122,126,127,128].
- Implementing standard protocols for integrated surveillance, which include coordinated monitoring of human cases, sentinel animals (horses, birds), vectors (mosquitoes, ticks), and integration of laboratory data, with rapid reporting between countries [129].
- Using digital platforms and web GIS systems to collect, store, and share epidemiological, entomological, and laboratory data, facilitating real-time visualization of risk and outbreaks across borders [130].
- Participating in European networks and projects (e.g., VectorNet, ECDC, MERMAIDS-ARBO), which provide infrastructure for standardized data exchange, validation of methods, and access to tools for risk analysis and mapping [131].
- Harmonizing diagnostic algorithms and case criteria to ensure data comparability and reduce reporting gaps between countries [36].
- Organizing regular meetings, simulation exercises, and After Action Reviews to identify strengths and gaps in collaboration and improve joint response to outbreaks [126].
- Promoting the exchange of expertise, resources, and good practices between institutions, including training and the development of common operating procedures [132].

## 6. Challenges and Opportunities

### 6.1. Challenges

The main challenges in implementing integrated surveillance of vector-borne zoonotic diseases, such as West Nile virus and Lyme disease, in the context of regional collaboration between Bulgaria, Hungary, and Serbia are underfunded surveillance programs, limited interdisciplinary collaboration, and a lack of public awareness, including low vaccination rates against tick-borne encephalitis.

Underfunded surveillance programs lead to incomplete geographical coverage and a lack of continuous monitoring of vectors and sentinel animals, as well as insufficient laboratory infrastructure for advanced molecular and serological diagnostics. This affects the capacity for early detection and rapid response to outbreaks, as highlighted in the recent literature on West Nile virus and tick-borne disease surveillance in the region [122,129,132].

Limited interdisciplinary collaboration is manifested by data fragmentation between the human, veterinary, and entomological sectors, the lack of common protocols and inter-institutional working groups, and difficulties in cross-border data exchange. Insufficient integration of surveillance reduces the effectiveness of outbreak prediction and control and has been highlighted as a major barrier to implementing the One Health approach [133,134].

Lack of public awareness, including low vaccination rates against tick-borne encephalitis, contributes to underdiagnosis, delayed presentation to health care providers, and the maintenance of an elevated risk of transmission. Information and education campaigns are insufficient, and access to vaccination remains limited, as highlighted in studies on the management and prevention of vector-borne diseases in Central and Eastern Europe [88,135].

These challenges require adequate funding, the development of integrated protocols, regional collaboration, and increased public education to increase the effectiveness of surveillance and control of vector-borne zoonotic diseases.

### 6.2. Opportunities

The main opportunities for improving integrated surveillance of vector-borne zoonotic diseases in the context of regional collaboration between Bulgaria, Hungary and Serbia are: leveraging the European Union’s Horizon programs to fund surveillance and research infrastructure, implementing ECDC initiatives for standardization, interoperability and data exchange, using big data and artificial intelligence for epidemiological prediction, and strengthening genomic sequencing networks as a legacy of the COVID-19 pandemic.

The Horizon programs enable the development of integrated One Health systems, with a focus on prioritizing zoonotic pathogens, creating common protocols and funding cross-border collaborations, including for Crimea–Congo, West Nile, Lyme and TBE, as prioritized by EFSA and ECDC [134,136]. ECDC initiatives facilitate the modernization of IT infrastructure, automation of epidemiological data processing, expansion of sentinel surveillance, and exploitation of alternative sources (electronic records, wastewater), with a focus on interoperability and continuous collaboration between countries [81,137].

Big data and artificial intelligence allow the integration of data from multiple sources (clinical, entomological, veterinary, environmental), the automation of epidemiological signal detection, and the development of predictive models for hotspot identification and outbreak anticipation, with real-time validation and visualization [138]. Genomic sequencing networks, strengthened in the context of COVID-19, provide capacity for WGS and molecular typing, facilitating the tracing of transmission lines, the identification of emerging strains, and the rapid exchange of data among regional laboratories [139].

**Figure 1:**
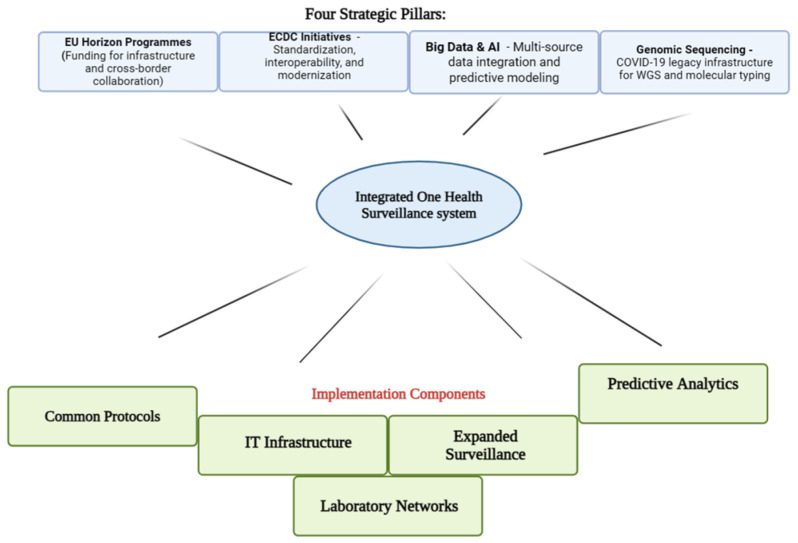
Flowchart showing how the strategic opportunities flow through integration and implementation to achieve enhanced surveillance outcomes for priority vector-borne diseases. Created in BioRender. Duduveche, A. (2025) https://BioRender.com/ybkt07j (accessed on 21 October 2025).

The integration of these tools and strategies, with the support of the EU and ECDC, will increase the capacity for early detection, rapid response, and effective control of vector-borne zoonotic diseases in the region (Figure 1).

## 7. Future Directions

The development of early warning systems combining entomological, climatic, and genomic data, using interoperable digital platforms and artificial intelligence algorithms for outbreak prediction, is essential for risk anticipation and rapid intervention [130]. The integration of these data requires standardization of collection processes and common protocols, as well as a scalable IT infrastructure for real-time processing [140].

The expansion of sentinel animal monitoring programs (birds, horses, rodents) for early detection of pathogens, with serological and molecular testing, allows for the identification of viral circulation before human cases appear and optimizes the epidemiological response [133,136]. These programs need to be coordinated across borders and integrated with vector and human case surveillance.

Integrating citizen science applications for participatory surveillance, including reporting of mosquito/tick sightings via smartphone and validation of data by laboratory, can expand geographic coverage and accelerate detection of invasive vectors or seasonal changes [16,90]. Linking these data with official surveillance systems increases epidemiological accuracy and utility.

Training the public health workforce in modeling, bioinformatics, and interpretation of complex data is vital for the effective functioning of early warning systems and for optimal use of integrated data [140]. Interdisciplinary training programs and collaboration with experts in climatology, entomology, and IT are recommended.

Advocacy for routine vaccination against tick-borne encephalitis in endemic regions is supported by strong evidence of reductions in TBE incidence following implementation of national programs, as observed in Austria [33]. Information campaigns and facilitating access to vaccination should be prioritized in high-risk areas.

## 8. Conclusions

The main conclusions regarding the surveillance of vector-borne infections in Romania are that traditional surveillance, based on human case reporting and seasonal vector monitoring, is insufficient to anticipate and control outbreaks in a dynamic epidemiological context marked by climate change, globalization, and urbanization. There is significant underreporting, especially in rural areas, data fragmentation between sectors (human, animal, vector), and limited genomic infrastructure, which reduces the capacity for early detection and rapid response.

Similar climate-driven shifts in vector distribution and disease transmission have been documented in neighboring Central and Southeastern European countries, including Hungary, Bulgaria, Serbia, and Italy. These shared trends suggest that Romania’s epidemiological challenges are part of a broader regional pattern, underscoring the need for coordinated, predictive surveillance strategies that extend beyond national borders.

Recommended predictive methodologies include establishing early warning systems that synthesize entomological, climatic, and genomic information, employing advanced mathematical models and artificial intelligence to forecast outbreaks and pinpoint hotspots. Enhancing sentinel animal monitoring programs, in conjunction with digital tools for participatory surveillance, is crucial for effective data collection and risk mapping, while training public health professionals in modeling and bioinformatics is essential for successful implementation.

Adopting these integrated, predictive, and digital approaches, with cross-border collaboration and EU funding, can transform Romania into a regional leader and build resilient systems to prepare for future vector-borne outbreaks.

## Figures and Tables

**Table 1:** Integrated synthesis of epidemiological trends, reservoirs and vectors, surveillance gaps, and priority One Health solutions for major vector-borne diseases in Romania.

Disease	Main Vectors in Romania	Key Reservoirs	Geographic Hotspots and Risk Areas	Human Epidemiological Signal (Last Decade)	Key Surveillance Gaps	Priority Solutions and Predictive Opportunities
West Nile virus (WNV)	*Culex pipiens* (dominant); *Aedes albopictus* (emerging)	Wild birds (herons, gulls, corvids); horses (sentinel)	Southeastern Romania, Danube Delta, Bucharest–Ilfov, urban and peri-urban areas	Recurrent seasonal transmission with major outbreaks (e.g., 2010, 2016, 2018); ongoing circulation	Fragmented human–animal–vector data; limited genomic surveillance; uneven regional reporting	Integrated One Health surveillance; systematic avian and equine sentinel programs; expanded genomic sequencing; climate-driven early warning models
Tick-borne encephalitis virus (TBEV)	*Ixodes ricinus*	Small mammals (rodents); wild ungulates (sentinel)	Carpathian and sub-Carpathian regions; forested and mountainous areas	Sporadic, likely underdiagnosed cases; absence of clear national trends	Low clinical awareness; limited diagnostics; lack of routine tick and wildlife surveillance	Targeted tick surveillance; seroprevalence studies; improved diagnostics; risk-based vaccination strategies
Lyme borreliosis	*Ixodes ricinus*	Rodents, deer, birds (dispersal hosts)	Nationwide, higher risk in forested, peri-urban, and recreational areas	Most frequently reported tick-borne disease; strong underreporting suspected	Heterogeneous diagnostics; incomplete reporting; limited integration of private laboratory data	Standardized diagnostic algorithms; mandatory reporting; integration of laboratory networks; enhanced tick monitoring
Crimean–Congo hemorrhagic fever virus (CCHFV)	*Hyalomma* spp. (potential/emerging)	Livestock (cattle, sheep); wild mammals	Southern and southeastern Romania (ecologically suitable areas)	No confirmed autochthonous human cases; potential emergence risk	Absence of systematic tick and animal surveillance; limited clinician preparedness	Proactive *Hyalomma* surveillance; livestock serology; clinician training; preparedness and rapid-response planning

## Data Availability

No new data were created or analyzed in this study. Data sharing is not applicable to this article.

## Abbreviations

The following abbreviations are used in this manuscript:

An	Anopheles
CCHF	Crimean–Congo Hemorrhagic Fever
COVID-19	Coronavirus disease 2019
DNA	Deoxyribonucleic acid
ECDC	European Centre for Disease Prevention and Control
EFSA	European Food Safety Authority
ELISA	Enzyme-Linked Immunosorbent Assay
EU/EEA	European Union/European Economic Area
GAM	Generalized Additive Model
GIS	Geographic Information System
IgG	Immunoglobulin G
IgM	Immunoglobulin M
INSP	National Institute of Public Health
IT	Information Technology
MaxEnt	Maximum Entropy
MLST	Multilocus Sequence Typing
NGS	Next-Generation Sequencing
NS3/NS5	Nonstructural proteins 3 and 5
PCR	Polymerase Chain Reaction
qPCR	Quantitative (real-time) PCR
RNA	Ribonucleic acid
SARIMA	Seasonal Autoregressive Integrated Moving Average
s.l.	sensu lato
spp	species
TBE	Tick-Borne Encephalitis
TBEV	Tick-Borne Encephalitis Virus
USUV	Usutu virus
WGS	Whole-Genome Sequencing
WNV	West Nile Virus
